# Targeting PfRh5 on Merozoites to Prevent Basigin Binding

**DOI:** 10.1186/1475-2875-11-S1-P124

**Published:** 2012-10-15

**Authors:** Konstantinos Papadopoulos

**Affiliations:** 1 Loyola University of Chicago; University of Glasgow; UK

## 

The goal of the research study was to shed light on a promising research area which may prove to be a safer more specific vaccine candidate; PfRH5 on merozoites. Erythrocyte invasion is paramount to the pathogenesis of *P. falciparum* requiring a series of extracellular recognition between the erythrocyte receptors and merozoites ligands. Of the known receptor-ligand interactions, one has been found essential to erythrocyte invasion in all *P. falciparum* strains; P. falciparum reticulocyte-binding protein homologue, PfRh5. This ligand binds to erythrocytes via basigin, an Ok blood group antigen and a type I integral membrane receptor consisting of many ligands. Basigin, also known as CD147, is a protein receptor on red blood cells, and a member of the immunoglobulin superfamily (Crosnier et al. 2011). It has further been found that a cysteine-rich protein called P. falciparum Rh5 interacting protein, PfRipr, forms a complex with PfRh5 on merozoites, where one study found antibodies to PfRipr1 to effectively inhibit merozoites attachment and invasion in erythrocytes (Chen et al. 2011).

The most effective targeting strategy seems to be preventing PfRh5 on merozoites from binding the receptor basigin on red blood cells (RBC). This receptor is essential for *Plasmodium falciparum* to enter RBC by binding basigin via PfRh5 protein on the malaria parasite. Basigin is a member of the immunoglobulin superfamily, a group of cell surface soluble proteins involved in the binding, adhesion, and recognition processes of cells. Since PfRH5 is specific to merozoites, a vaccine based on PfRh5 would not affect RBCs or other parts of the body, likely not initiating an auto-immune response. This presents the best opportunity due to the specificity of PfRH5 which makes it a “safe” and effective vaccine targeting strategy.

Due to the specificity of the attack method used by *P. falciparum*, it seems evident that the vaccine must target a specific receptor of one of the parasites many stages. The focus of the study is to shed light on this newly found research and to create a vaccine in the future that develops antibodies against the parasite ligand of Basigin, Rh5, since PfRh5 protein on the parasite binds to basigin. Due to the need of merozoites to enter and replicate in RBC, this seems like the most plausible vaccine development. Since humans are both the carriers and “spreaders” to mosquitos, it is paramount to find a vaccine for humans to stop the spread of gametocytes to mosquitos which would prevent sporozoites formation and the re-infection of other humans. The author believes that developing antibodies against the parasite ligand for basigin will prevent merozoites invasion into erythrocytes.

